# Duplication and Loss of Function of Genes Encoding RNA Polymerase III Subunit C4 Causes Hybrid Incompatibility in Rice

**DOI:** 10.1534/g3.117.043943

**Published:** 2017-06-07

**Authors:** Giao Ngoc Nguyen, Yoshiyuki Yamagata, Yuko Shigematsu, Miyako Watanabe, Yuta Miyazaki, Kazuyuki Doi, Kosuke Tashiro, Satoru Kuhara, Hiroyuki Kanamori, Jianzhong Wu, Takashi Matsumoto, Hideshi Yasui, Atsushi Yoshimura

**Affiliations:** * Plant Breeding Laboratory, Faculty of Agriculture, Kyushu University, Fukuoka 812-8581, Japan; † Molecular Gene Technics Laboratory, Faculty of Agriculture, Kyushu University, Fukuoka 812-8581, Japan; ‡ Agrogenomics Research Center, National Institute of Agrobiological Sciences, Tsukuba, Ibaraki 305-8634, Japan

**Keywords:** reproductive barrier, genetic drift, neutral gene, nonfunctionalization, hybrid sterility

## Abstract

Reproductive barriers are commonly observed in both animals and plants, in which they maintain species integrity and contribute to speciation. This report shows that a combination of loss-of-function alleles at two duplicated loci, *DUPLICATED GAMETOPHYTIC STERILITY 1* (*DGS1*) on chromosome 4 and *DGS2* on chromosome 7, causes pollen sterility in hybrid progeny derived from an interspecific cross between cultivated rice, *Oryza sativa*, and an Asian annual wild rice, *O. nivara*. Male gametes carrying the *DGS1* allele from *O. nivara* (*DGS1-nivara^s^*) and the *DGS2* allele from *O. sativa* (*DGS2-T65^s^*) were sterile, but female gametes carrying the same genotype were fertile. We isolated the causal gene, which encodes a protein homologous to DNA-dependent RNA polymerase (RNAP) III subunit C4 (RPC4). RPC4 facilitates the transcription of 5S rRNAs and tRNAs. The loss-of-function alleles at *DGS1-nivara^s^* and *DGS2-T65^s^* were caused by weak or nonexpression of *RPC4* and an absence of *RPC4*, respectively. Phylogenetic analysis demonstrated that gene duplication of *RPC4* at *DGS1* and *DGS2* was a recent event that occurred after divergence of the ancestral population of *Oryza* from other Poaceae or during diversification of AA-genome species.

Hybrid incompatibility is a phenomenon of maladaptive traits such as inviability, weakness, and sterility in hybrids between two diverged populations or species and in their progeny. Hybrid incompatibility is a common feature of a wide range of organisms (Coyne and Orr 2004). It plays a vital role in intrinsic postzygotic reproductive isolation, which conserves species integrity and uniformity and contributes to speciation by limiting gene flow within and between species (Lynch and Force 2000; Taylor *et al.* 2001).

The genetic basis of hybrid incompatibility has been analyzed in many plants and animals (Coyne and Orr 2004; Bomblies and Weigel 2007; Rieseberg and Blackman 2010). Epistasis of nuclear-encoded genes for hybrid incompatibility, known as Bateson–Dobzhansky–Muller (BDM) incompatibility, is a major genetic phenomenon in which interaction of alleles at different loci leads to maladaptive traits in hybrids derived from crosses between two diverged populations or species (Coyne and Orr 2004; Noor and Feder 2006). Models suggest that genes causing hybrid incompatibility are maintained in species with a compatible genetic background. Even though the BDM model is simple and universal, the interaction of independently evolved genes from two diverged populations leads to conflicts in a variety of biological processes, including molecular interaction related to DNA binding (Brideau *et al.* 2006), intercellular protein trafficking through plasmodesmata (Long *et al.* 2008), the endoplasmic reticulum stress response (Yang *et al.* 2012), and autoimmune responses caused by aberrant signal transduction systems (Bomblies and Weigel 2007; Yamamoto *et al.* 2010; Chen *et al.* 2014).

Gene duplication is considered to be a main driver of evolution because it supplies functional redundancy, which allows functional changes in one or more of the duplicate gene copies while keeping the original function of the ancestral gene, which may be essential for survival (Ohno 1970; Lynch and Conery 2000; Moore and Purugganan 2005). Duplicated genes are suggested to meet three functional fates: acquisition of a novel and beneficial function (neofunctionalization), specialization of an ancestral gene’s function across duplicates (subfunctionalization), and silencing or loss of function through degenerative mutations (nonfunctionalization). Although duplicate genes arise at a very high rate, the vast majority of duplicate genes rapidly undergo nonfunctionalization within a few million years (Lynch and Conery 2000). Since silencing or loss of function at unlinked gene duplicates are stochastic processes for young duplicate copies (designated *A_1_A_1_A_2_A_2_*), the probabilities of generating genotype *A_1_A_1_a_2_a_2_* in one species and genotype *a_1_a_1_A_2_A_2_* in the other species are assumed to be identical. These processes for gene duplications and subsequent losses at the reciprocal loci in each species [hereafter designated as gene duplication and reciprocal loss (GDRL)] result in positional variation of functional genes among diverged populations or species, and zygotes (*a_1_a_1_a_2_a_2_*) or gametes (*a_1_a_2_*) carrying the double null genes exhibit maladaptive phenotypes in hybrid progeny (Lynch and Force 2000). Hybrid incompatibility caused by the GDRL process can be considered as a kind of BDM incompatibility. Molecular cloning studies have revealed the zygotic type of BDM incompatibility caused by GDRL in a histidinol-phosphate aminotransferase gene, which results in lack of free histidine in hybrid embryos (Bikard *et al.* 2009); gametophytic BDM incompatibility caused by the GDRL process affecting nuclear-encoded mitochondrial ribosomal protein in pollen (Yamagata *et al.* 2010; Win *et al.* 2011); and GDRL leading to failure of pollen tube germination (Mizuta *et al.* 2010).

Eukaryotes synthesize RNA by multi-subunit protein complexes, the RNAPs: designated PolI for 45S preribosomal RNA, PolII for mRNA and noncoding RNA, and PolIII for tRNA and 5S rRNA (Vannini and Cramer 2012). PolI, PolII, and PolIII have similar structures with shared ancestral origin from 12 subunits (Rpo1–12) of archaean RNAP (Ream *et al.* 2009). In addition to the canonical PolII, plants have acquired two plant-specific multi-subunit RNAP homologs, PolIV and PolV, for RNA-directed DNA methylation and transcriptional silencing via neofunctionalization of gene duplicates for PolII components (Onodera *et al.* 2005; Haag and Pikaard 2011). Different angiosperm groups have experienced different lineage-specific gene duplication of several RNAP subunits, implying independent functional divergences of RNAP components (Wang and Ma. 2015). Neofunctionalization for RNAPs is likely to have resulted in acquisition of specific biological processes in plants. However, it has not been fully understood whether nonfunctionalization of gene duplicates for RNAP members drives diversification of plant species.

Here, we report the identification and cloning of the genes for F_1_ pollen sterility in an interspecific hybrid between the cultivated species *Oryza sativa* L. and the wild species *O. nivara* Sharma et Shastry. We show that the pollen sterility in the F_1_ hybrid is due to the epistatic interaction of two duplicated loci on chromosomes 4 and 7. Map-based cloning revealed that the two genes encode a putative RNAP III subunit C4 (RPC4), which is known to be involved in the transcription of 5S rRNAs and tRNAs.

## Materials and Methods

### Plant materials

Accessions of *O. nivara* Sharma et Shastry IRGC105715, originating from Cambodia, were kindly provided by the International Rice Research Institute, Manila, the Philippines. *O. sativa* L. ssp. *japonica* “Taichung 65” (T65) was crossed with pollen of IRGC105715 to produce F_1_ plants with T65 cytoplasm. Near-isogenic lines were developed by performing repeated backcrosses with T65 as the male parent (Supplemental Material, Figure S1 in File S1). For the linkage mapping of *DGS1* and *DGS2* (see *Results*), two kinds of BC_4_F_3_ plants were selected from the BC_4_F_2_ population: (1) plants heterozygous at *RM16862* (linked to *DGS1*) on chromosome 4 and homozygous for the T65 allele at *M41_STS* (linked to *DGS2*) on chromosome 7 (later designated the *DGS2-T65^s^* allele), and (2) plants homozygous for the *O. nivara* allele at *RM16862* (later designated the *DGS1-nivara^s^* allele) and heterozygous at *M41_STS*. These plants were grown to develop BC_4_F_5_ populations for high-resolution mapping. For genetic analysis of the epistatic interaction between *DGS1* and *DGS2*, we used two BC_4_F_3_ populations derived from BC_4_F_2_ plants heterozygous for *RM16862* and *M41_STS*.

### Observation of mature pollen grains

Panicles at the preflowering stage were collected and fixed in solution containing 4% (w/v) paraformaldehyde, 0.25% (w/v) glutaraldehyde, 0.02% (v/v) Triton X-100, and 100 mM sodium phosphate (pH 7.5) at 4° for 24 hr. The panicles were rinsed in 100 mM sodium phosphate buffer and stored in the same buffer containing 0.1% (w/v) sodium azide (NaN_3_). Pollen grains were stained in hematoxylin solution according to Chang and Neuffer (1989) and observed under a light microscope.

### Evaluation of pollen fertility

Spikelets at the flowering stage were fixed in 70% ethanol. Pollen grains from all six anthers of a single spikelet were released onto a glass slide and stained with 1% I_2_–KI solution. Approximately 200 grains were observed and evaluated for fertility under an Axioplan light microscope (Zeiss, Jena, Germany). Pollen germination was tested *in vitro* by releasing pollen grains from dehisced anthers immediately onto germination gel containing 15% sucrose, 0.01% H_3_BO_3_, 0.03% CaCl_2_, and 0.6% Gelrite (pH 7.2) (Wako, Osaka, Japan) dropped on a slide glass. The slide glasses were incubated at room temperature for 5 min, and pollen germination was observed under a light microscope. Images were processed in Photoshop software (Adobe, San Jose, CA).

### Linkage mapping

A total of 111 simple sequence repeat (SSR) markers were used for genome-wide genotyping of the BC_4_F_1_ pollen-sterile plants to find regions of chromosomal introgression from *O. nivara* that were involved in pollen sterility in the T65 genetic background (Table S1 in File S2). Three pairs of primers for SSR markers WGS76, WGS1, and WG1 were newly designed in this study in SSRIT software (Temnykh *et al.* 2001). Five SSR markers (*RM471* and *RM1359* on chromosome 4 and *RM6652*, *RM1353*, and *RM6081* on chromosome 7) were used for linkage analysis in the BC_4_F_3_ population (Table S1 in File S2). Total genomic DNA was extracted according to the method of Dellaporta *et al.* (1983) with minor modifications. Each 15 μl reaction mixture consisted of 50 mM KCl, 10 mM Tris (pH 9.0), 1.5 mM MgCl_2_, 200 μM dNTPs, 0.2 μM primers, 0.75 units of *Taq* polymerase (Takara, Otsu, Japan), and ∼10 ng genomic DNA template. PCR was performed in a GeneAmp PCR System 9700 (Applied Biosystems, Foster City, CA). The cycling profile was an initial denaturation at 95° for 5 min; 35 cycles of 95° for 30 sec, 55° for 30 sec, 72° for 40 sec; and a final elongation step at 72° for 7 min. Amplified products were electrophoresed in 4% agarose gel in 0.5× TBE buffer. MAPMAKER/EXP v. 3.0 software (Lander *et al.* 1987) was used to construct a linkage map using Kosambi’s mapping function.

### Map-based cloning

For the high-resolution mapping of *DGS1* and *DGS2*, genotypes of recombinants were determined by additional SSR and sequence-tagged site (STS) markers shown in Table S2 in File S2. PCR primers for the STS markers *M45_STS*, *M41_STS*, and *M23_STS* were designed from the rice reference sequence of Nipponbare in SSRIT (Temnykh *et al.* 2001), and amplified PCR products from the T65 and *O. nivara* genomes were separated by electrophoresis in 4% agarose gel. For the BAC clone screening by PCR to investigate the genomic structures of T65 and *O. nivara*, *RM16862* and *RM16867* were used for *DGS1* and *M45_STS* and *M41_STS* were used for *DGS2*. The obtained BAC clones GN0025A01, GN0028G14, and GN0031H20 (T65) and IRGC105715_0023B24 (*O. nivara*) were sequenced by next-generation sequencing using Roche/454 GS-FLX Titanium sequencing (Roche, Basel, Switzerland) and *de novo* assembly in GS *De novo* Assembler software (Roche). Shotgun sequencing by the Sanger sequencing method was conducted for the *O. nivara* BAC clone IRGC105715_0046I04.

A complementation test was conducted as described by Yamagata *et al.* (2010). In brief, *Eco*RI-digested genomic fragments containing predicted *RPC4* genes were cloned into the Ti-plasmid binary vector pPZP2H-lac (Fuse *et al.* 2001), which was transformed into *DGS1* semisterile plants (*T^+^/N^s^*|*T^s^/T^s^*) and *DGS2* semisterile plants (*N^s^/N^s^*|*T^s^/N^+^*) via *Agrobacterium*-mediated transformation (The two semisterile plant types were designated by which of the two loci was heterozygous for fertile and sterile alleles. In each case, the other locus was homozygous for the sterile allele.). Complementation of pollen fertility by the transgenes was examined by the segregation ratios of genotypes at *DGS1* and *DGS2* using SSR markers tightly linked to *DGS1* (marker *RM471*) and *DGS2* (marker *RM6574*) in the T_1_ generation.

Copy numbers of the transgenes inserted in the genome of the T_0_ and T_1_ plants were estimated by quantitative PCR in a MX3000P QPCR system (Agilent Technologies, Santa Clara, CA) using QuantiTect SYBR Green PCR Kits (QIAGEN, Venlo, The Netherlands). The standard curve was based on the amplification of the CaMV 35S promoter region using primers 5′-CGT AAG GGA TGA CGC ACA ATC C-3′ and 5′-CGA GAT TCT TCG CCC TCC GA-3′. Amplification of a single-copy region of rice genomic chromosome 8 DNA using the primers 5′-GGA CTG GAC AGA TTG AGA GTG-3′ and 5′-AAC GCC GAA CAA GCC CTT ACA-3′ was used as the internal control. The thermal cycling profile was an initial denaturation at 95° for 15 min, followed by 35 cycles of 95° for 30 sec, 55° for 30 sec, and 72° for 30 sec.

### Gene structure and expression analysis

To investigate the structure of the transcript of the causal gene(s) of *DGS1* and *DGS2*, 5′- and 3′-rapid amplification of cDNA ends (RACE) was conducted using the SMARTer RACE cDNA synthesis kit (Clontech, Mountain View, CA). Total RNA was extracted from anthers using the mirVana miRNA isolation kit (Ambion, Carlsbad, CA). Amplification products were cloned into the pGEM-T Easy vector (Promega, Madison, WI) and introduced into DH5α competent cells. The plasmids were sequenced with a BigDye Terminator v. 3.1 cycle sequencing kit and analyzed on a 3130xl Genetics Analyzer (Applied Biosystems). The sequencing data were aligned to the genomic sequences of T65 and *O. nivara* from BAC clones in Sequencher software (Gene Codes, Ann Arbor, MI).

For expression analysis of *DGS1* and *DGS2*, 20 ng/μl of total RNA was used for quantitative reverse-transcription polymerase chain reaction (qRT-PCR) analysis using the forward primer 5′-ATG TCT CTC CGG GTT CAA ATT GC-3′ and the reverse primer 5′-TTA CGC TTC CAT CTT GTC GAA AGA ATC C-3′. The SuperScript III Platinum SYBR Green One-Step qRT-PCR Kit (Invitrogen, Carlsbad, CA) was used for real-time reactions as described previously (Nguyen *et al.* 2010). Rice *Ub-CEP52-1* encoding ubiquitin-fused 60S ribosomal protein L40-1 (*Os03g0234200*), amplified by the forward primer 5′-CTG TCA ACT GCC GCA AGA AG-3′ and the reverse primer 5′-GGC GAG TGA CGC TCT AGT TC-3′, was used as an internal control for the qRT-PCR.

### Promoter–GUS assay

To analyze the spatial expression patterns of *RPC4*, a 2.4 kb PCR fragment upstream of the coding sequence was amplified from T65 BAC clone GN0028G14 using the forward primer 5′-CAC CGG TAG GGG AAG GAC ATG A-3′ and the reverse primer 5′-ACA AGA AAA GAA GCA CAA ATC CTG CG-3′ and cloned into the pENTR/D-TOPO vector (Invitrogen). Plasmids were sequenced to confirm that no mutation had occurred during the PCR amplification. The cloned endogenous promoter sequence was fused to a promoterless *GUS* gene derived from the pGWB3 Gateway-system destination vector (Nakagawa *et al.* 2007) by using LR Clonase (Invitrogen). The final construct was transformed into T65 using *Agrobacterium*-mediated transformation. Spikelets of the T_0_ plants at the mature stage of pollen development were collected and stained for *in situ* determination of *GUS* activity, as described by Yamagata *et al.* (2010).

### Data availability

Strains are available upon request. File S1 contains all of the supplemental figures and methods. All genotype data are presented in the supplemental tables (File S2). Sequence data have been deposited in the DNA Data Bank of Japan for *RPC4* from the *DGS1-T65^+^* allele (AB758279); *RPC4* from the *DGS1-nivara^s^* allele (AB758280); *RPC4a* (AB758281), *RPC4b* (AB758282), *RPC4c* (AB758283), *RPC4d* (AB758284), and *RPC4e* (AB758285) from the *DGS2-nivara^+^* allele; and BAC sequences of GN0028G14 for *DGS1-T65^+^* (LC060740), IRGC105715_0023B24 for *DGS1-nivara^s^* (LC060741), GN0031H20 for *DGS2-T65^s^* (LC060743), and IRGC105715_0046I04 for *DGS2-nivara^+^* (LC060742).

## Results

### Identification of genetic factors on chromosomes 4 and 7

**Figure 1:**
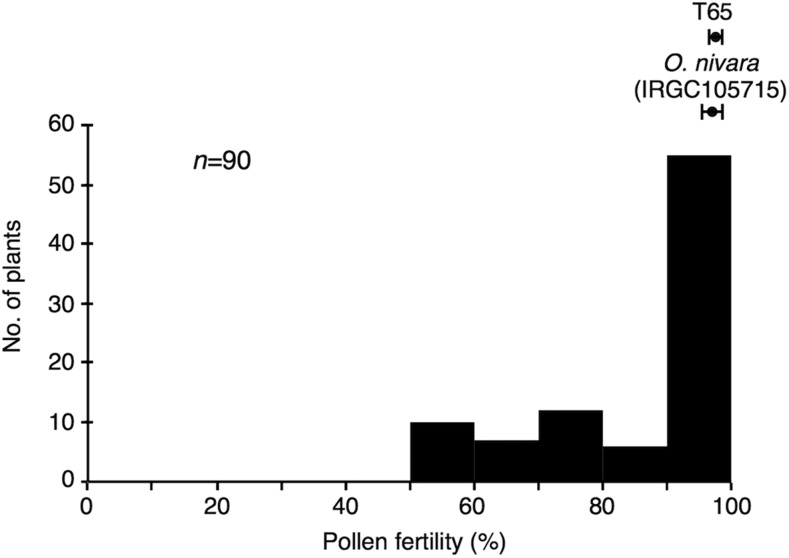
Frequency distribution of pollen fertility in the BC_4_F_3_ populations derived from a cross between T65 (*O. sativa*) and IRGC105715 (*O. nivara*). The BC_4_F_1_ parents were heterozygous for markers on chromosomes 4 (at *RM471* and *RM1359*) and 7 (at *RM6652*, *RM1353*, and *RM6081*) (Figure S1 in File S1). The pollen fertility of each parent is indicated at the top; error bars indicate SEs (*n* = 3). No., number.

In developing introgression lines of *O. nivara* genomic segments in a T65 genetic background, we produced a BC_4_F_1_ population by several generations of backcrossing with T65 as the male parent. In the progeny of a single BC_4_F_2_ population (“BC_4_F_2_ 42” in Figure S1 in File S1), we observed a kind of trimodal distribution with pollen fertility ranging from 50 to 100% (Figure 1) in the two BC_4_F_3_ populations derived from two BC_4_F_2_ plants heterozygous for markers on chromosomes 4 (at *RM471* and *RM1359*) and 7 (at *RM6652*, *RM1353*, and *RM6081*) (Figure S1 in File S1). We assumed that this pollen sterility was governed by epistatic interaction of duplicated sterile genes at two segregating loci, known as the gametophytic type of BDM incompatibility model, in which pollen sterility levels of 50, 75, and 100% are expected to segregate in a 2:3:7 ratio (Figure S2 in File S1), because we have seen a similar pollen fertility distribution in the hybrid pollen sterility governed by the duplicated loci *S27* and *S28* in *O. sativa* and *O. glumaepatula* hybrid (Yamagata *et al.* 2010). The observed frequency distribution of pollen fertility in the BC_4_F_3_ populations was similar to the distribution predicted by the model (Figure 1 and Figure S2 in File S1). Genotyping at *RM471* on chromosome 4 and *RM6081* on chromosome 7 showed that genotypes in the BC_4_F_3_ populations fit the expected ratio for the gametophytic type of BDM incompatibility model of 1:2:1:1:3:2:0:1:1, which assumes that pollen grains carrying the *O. nivara* allele at *RM471* and the T65 allele at *RM6081* are sterile (Figure S2 in File S1, Table S3 in File S2 and Table 1) [hereafter, T65 and *O. nivara* alleles are indicated by *T* and *N*, respectively, and the letters to the left and right of the vertical bar indicate genotypes at *RM471* (*DGS1*) and *RM6081* (*DGS2*), respectively; sterile alleles, *i.e.*, the *O. nivara* allele at *RM471* (*DGS1)* and the T65 allele at *RM6081* (*DGS2*), are indicated with a superscript of *s*; and normal alleles, *i.e.*, the T65 allele at *DGS1* and the *O. nivara* allele at *DGS2*, are indicated with a superscript of *+*]. In particular, no plants homozygous for *O. nivara* at *RM471* and homozygous for T65 at *RM6081* (genotype *N^s^/N^s^*|*T^s^/T^s^*; Table 1) were observed among 90 individuals of this BC_4_F_3_ population, proving further evidence that pollen carrying *N^s^*|*T^s^* was sterile and therefore not transmitted to the progeny.

**Table 1:** Epistatic interaction observed between genotypes at *RM6081* and *RM471* fitted to a gametophytic BDM incompatibility model in BC_4_F_3_ populations derived from a cross between T65 (*O. sativa*) and IRGC105715 (*O. nivara*)

Genotype		Expected Ratio*^a^*	Number of Individuals		χ^2^*^b^*
*RM471* (*DGS1*)	*RM6081* (*DGS2*)		Expected	Observed	
*T/T**^c^*	*T/T*	1/12	7.5	9	0.3
	*T/N*	2/12	15	14	0.07
	*N/N*	1/12	7.5	9	0.3
*T/N*	*T/T*	1/12	7.5	10	1.7
	*T/N*	3/12	22.5	18	0.9
	*N/N*	2/12	15	15	7.5
*N/N*	*T/T*	0/12	0	0	—
	*T/N*	1/12	7.5	7	0.03
	*N/N*	1/12	7.5	8	0.03
Total		1	90	90	2.46
*P*					0.92

a Expected ratio based on the hypothesis that *DGS1* and *DGS2* segregate independently, and that pollen of genotype *N*|*T* is sterile. See Figure S2 in File S1 for further explanation.

b Each value in the column contains rounding error.

c *T/T*, homozygous for T65 allele; *T/N*, heterozygous; and *N/N*, homozygous for *O. nivara* allele at the indicated marker locus.

### Identification of DGS1 and DGS2

**Figure 2:**
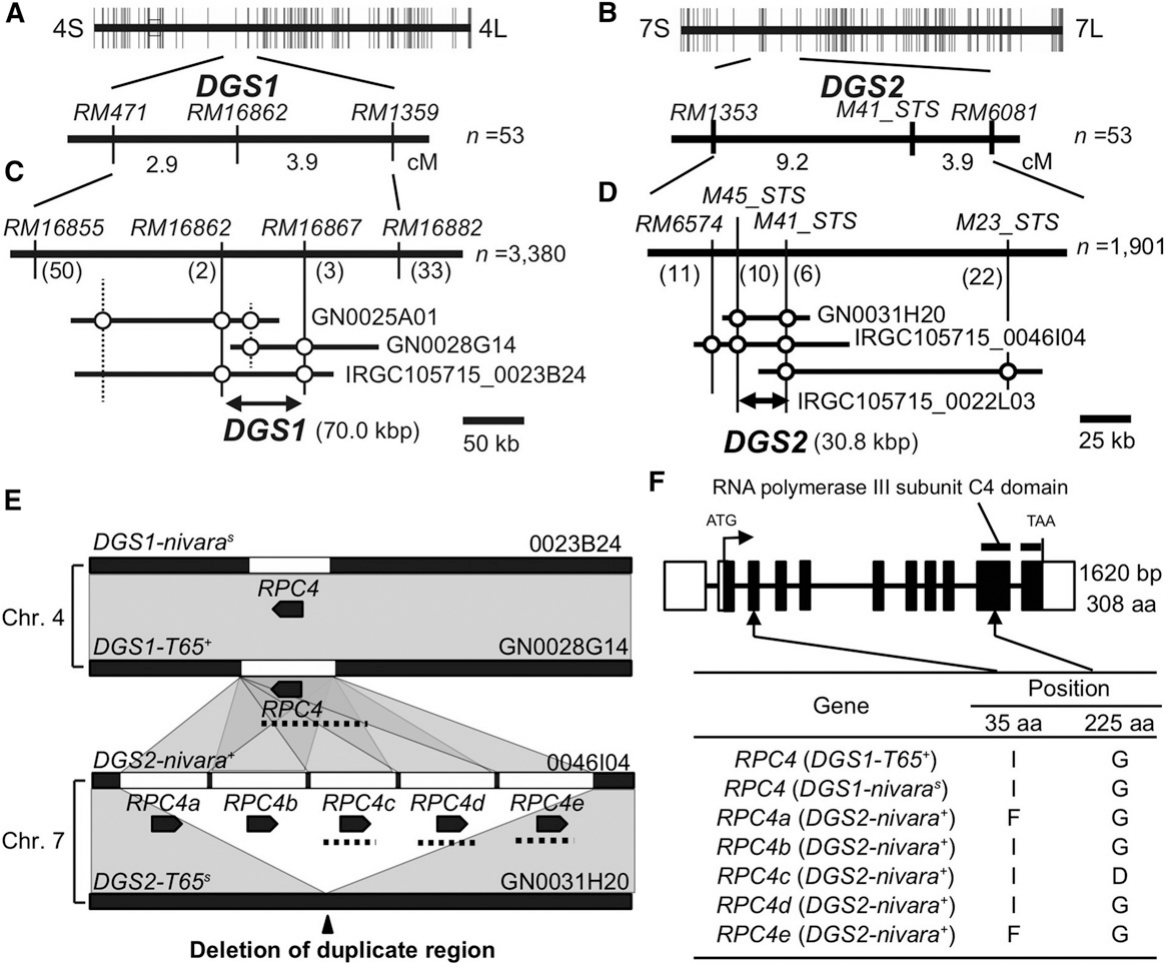
Linkage mapping and map-based cloning of *DGS1* and *DGS2*. (A and B) Linkage mapping of (A) *DGS1* and (B) *DGS2*. (C and D) High-resolution mapping of (C) *DGS1* and (D) *DGS2*. The number in parentheses beneath each marker represents the number of recombinants between that marker and the *DGS* gene. (E) Diagrams of the BAC clones in the *DGS1* and *DGS2* regions of T65 and *O. nivara*. Segmental duplications (white boxes) were found in the *DGS1-T65^+^*, *DGS1-nivara^s^*, and *DGS2-nivara^+^* alleles, but not in the *DGS2-T65^s^* allele. Black pentagons represent the predicted *RPC4* genes. Dotted lines represent the *Eco*RI fragments used for the transgenic complementation tests. Homologous genomic regions are connected by translucent gray areas. Black arrowhead indicates site where segmental duplication was not found or deleted in *DGS2-T65^s^*. (F) Structure of *RPC4*. White boxes represent 5′- and 3′-untranslated regions. Black boxes indicate coding sequences within exons. Arrows indicate the positions of amino acid polymorphisms among the copies of *RPC4*. Horizontal bars indicate position of the RNA polymerase III subunit C4 domain. BAC, bacterial artificial chromosome; Chr., chromosome.

We conducted linkage analysis to identify the postulated genetic factors on chromosomes 4 and 7. For linkage mapping of the genetic factor on chromosome 4, we selected the BC_4_F_2_ individual that was heterozygous on chromosome 4 and homozygous for the T65 allele on chromosome 7 (*T^+^/N^s^*|*T^s^/T^s^*) to produce a BC_4_F_3_ population segregating only for the segment on chromosome 4. Pollen-fertile and semisterile plants segregated in a ratio of 24:29 in the BC_4_F_3_ population (Table S4 in File S2 and Table 2). All homozygotes for the T65 allele at *RM16862* (*T^+^/T^+^*|*T^s^/T^s^*) were fertile and all heterozygotes (*T^+^/N^s^*|*T^s^/T^s^*) were pollen semisterile. As in previous analyses, no homozygotes for the *O. nivara* allele on chromosome 4 (*N^s^/N^s^*|*T^s^/T^s^*) were observed. These data suggest that sterility in this population was governed by a single gametophytically-acting gene tightly linked to *RM16862* on chromosome 4, which induced selective abortion or sterility of pollen grains carrying the *O. nivara* allele when the chromosomal segment on chromosome 7 was fixed for the T65 allele. We designated this locus on chromosome 4 as *DGS1*. Linkage analysis showed that *DGS1* cosegregated with *RM16862* and was located between *RM471* and *RM1359* with map distances of 2.9 and 3.9 cM, respectively (Figure 2A). The observed segregation distortion of genotypes in the BC_4_F_3_ population would be the expected result of gametophytic sterility of pollen grains carrying the *DGS1-nivara^s^* allele and the *DGS2-T65^s^* allele (pollen genotype *N^s^*|*T^s^*) in the semisterile BC_4_F_2_ plants.

**Table 2:** Linkage analysis of pollen fertility in a BC_4_F_3_ population derived from a BC_4_F_2_ plant heterozygous at *RM16862* (chromosome 4) and homozygous for the T65 allele at *M41_STS* (chromosome 7)

Pollen Fertility	Genotypes at *RM16862**^a^*		
	*T/T*	*T/N*	*N/N*
Fertile	24	0	0
Semisterile	0	29	0

a *T/T*, homozygous for T65 allele; *T/N*, heterozygous; and *N/N*, homozygous for *O. nivara* allele.

For linkage mapping of the genetic factor on chromosome 7, we took a similar approach. We developed three BC_4_F_3_ populations from three BC_4_F_2_ plants, which were homozygous for *O. nivara* at *RM471* on chromosome 4 and heterozygous at *RM6081* on chromosome 7 (*N^s^/N^s^*|*T^s^/N^+^*) in the BC_4_F_2_ 42 population (Figure S1 in File S1), to obtain a population segregating only for the segment on chromosome 7. In the BC_4_F_3_ population, fertile and semisterile plants segregated in a 25:28 ratio (Table S5 in File S2 and Table 3). All of the fertile plants were homozygous for the *O. nivara* allele at *M41_STS* (*N^s^/N^s^*|*N^+^/N^+^*), and all of the semisterile plants were heterozygous (*N^s^/N^s^*|*T^s^/N^+^*); no homozygotes for the T65 allele (*N^s^/N^s^*|*T^s^/T^s^*) were observed. As with *DGS1*, we considered that the semisterility and segregation ratios were caused by gametophytic sterility of pollen grains carrying the T65 allele of a locus tightly linked to *M41_STS* (*N^s^*|*T^s^* genotype). We named this locus *DGS2*. *DGS2* was mapped between *RM1353* (9.2 cM) and *RM6081* (3.9 cM) (Figure 2B).

**Table 3:** Linkage analysis of pollen fertility in BC_4_F_3_ populations derived from BC_4_F_2_ plants homozygous for the *O. nivara* allele at *RM16862* (chromosome 4) and heterozygous at *M41_STS* (chromosome 7)

Pollen Fertility	Genotype at *M41_STS**^a^*		
	*T/T*	*T/N*	*N/N*
Fertile	0	0	25
Semisterile	0	28	0

a *T/T*, homozygous for T65 allele; *T/N*, heterozygous; and *N/N*, homozygous for *O. nivara* allele.

### Characterization of sterility caused by DGS1 and DGS2

**Figure 3:**
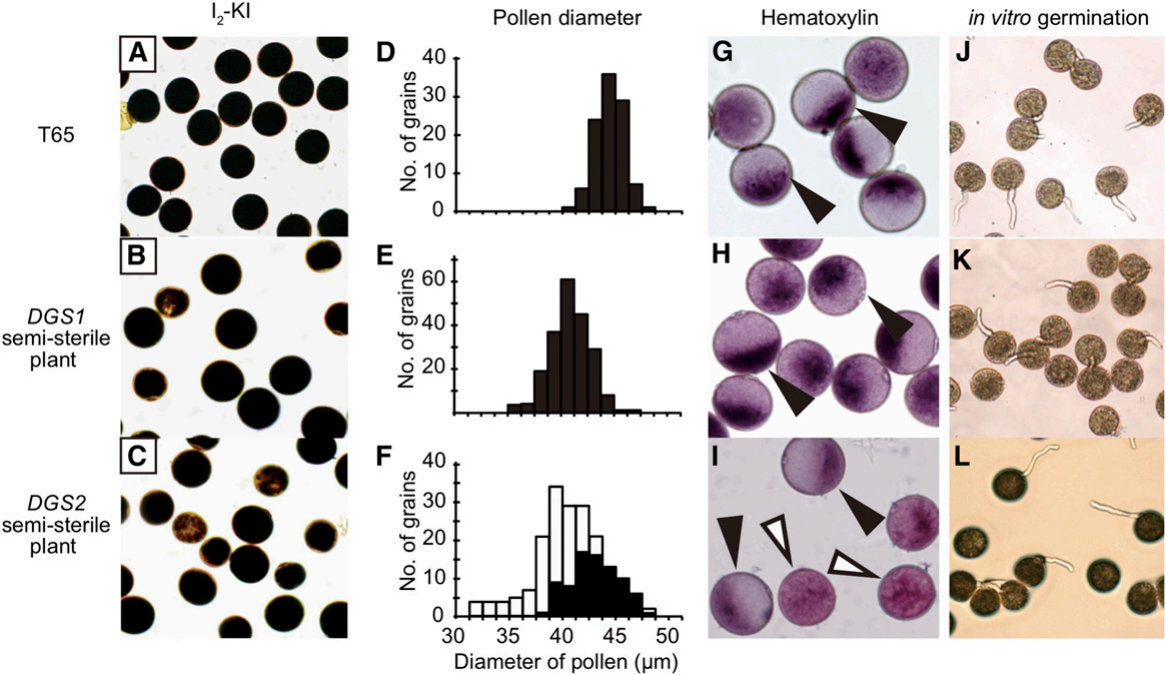
Characterization of sterility caused by *DGS1* and *DGS2*. (A–C) Mature pollen stained with I_2_–KI. (D–F) Frequency distribution of pollen grain diameter at the preflowering stage. Black and white bars indicate numbers of glycosylated and nonglycosylated pollen grains, respectively. (G–I) Pollen grains stained by hematoxylin at the preflowering stage. Black arrowheads, glycosylated grains; white arrowheads, nonglycosylated grains. (J–L) *In vitro* pollen germination test. No., number.

**Figure 4:**
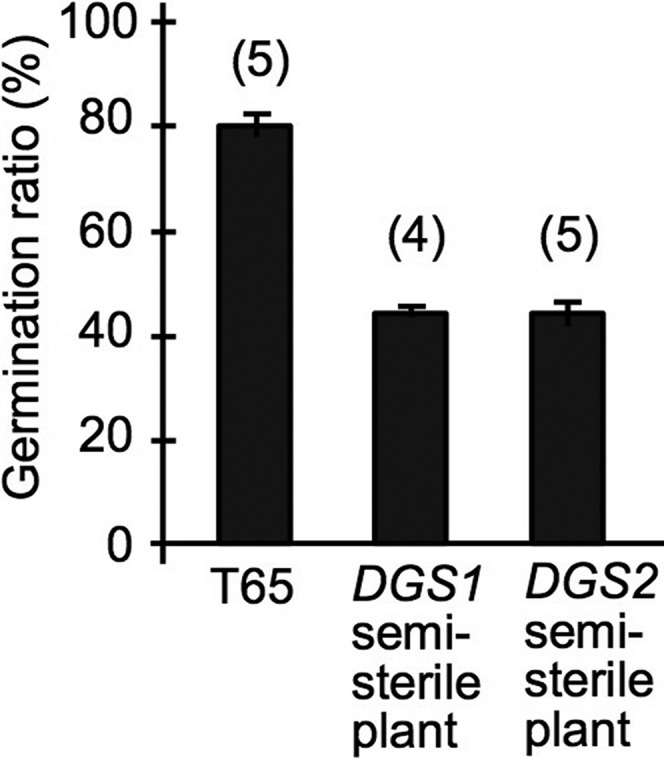
Pollen germination *in vitro*. Numbers in parentheses indicate numbers of plant replicates. Error bars indicate SEs in replications shown in parenthesis.

We investigated abnormalities induced by the epistatic interaction between *DGS1* and *DGS2* in postmeiotic development by observing the phenotypes of T65, BC_4_F_5_ plants carrying the *T^+^/N^s^*|*T^s^/T^s^* genotype (*DGS1* semisterile plants), and BC_4_F_5_ plants carrying the *N^s^/N^s^*|*T^s^/N^+^* genotype (*DGS2* semisterile plants). In each of the two semisterile plant types, 50% pollen sterility was expected because of the sterility of pollen grains carrying the *N^s^*|*T^s^* genotype. No abnormalities were observed in male gametophytes during the tetrad, unicellular, bicellular, or tricellular stages among T65, *DGS1* semisterile plants, or *DGS2* semisterile plants (data not shown). At the mature pollen stage, which occurs a few days before anthesis, almost all pollen grains stained well with I_2_–KI (Figure 3, A–C). Smaller pollen grains began to appear, but it was technically difficult to count their frequency because the size distribution was continuous, so two categories could not be clearly distinguished (Figure 3, D–F). At 1 hr before flowering (preflowering stage), most of pollen grains had begun glycosylation of starch in T65 and the *DGS1* semisterile plants (Figure 3, G–H), whereas only half of the pollen grains (91 glycosylated/191 grains in total) in the *DGS2* semisterile plants showed glycosylation (Figure 3I). At the flowering stage, 44.3 ± 1.6% of pollen grains from the *DGS1* semisterile plants and 44.4 ± 3.5% of those from the *DGS2* semisterile plants germinated *in vitro*, whereas 80.3 ± 4.5% of the T65 pollen grains germinated (Figure 3, J–L and Figure 4). Thus, half of the pollen grains from the *DGS1* and *DGS2* semisterile plants appeared to have lost their fertilization ability. These data demonstrate that for the *DGS1* and *DGS2* semisterile plants, the pollen germination and glycosylation stages, respectively, were the critical stages at which fertile and sterile pollen grains became evident.

### Map-based cloning

We conducted high-resolution mapping of *DGS1* using 3380 plants from BC_4_F_5_ populations derived from *DGS1* semisterile plants (Figure 2C). *DGS1* was mapped to a 70 kb genomic region between *RM16862* and *RM16867* in the reference sequence of Nipponbare, with two recombinants between *RM16862* and *DGS1* and three between *RM16867* and *DGS1* (Table S6 in File S2). In the high-resolution mapping of *DGS2* using 1901 plants of BC_4_F_5_ populations derived from *DGS2* semisterile plants, we located *DGS2* within a 30.8 kb genomic region of Nipponbare between *M45_STS* and *M41_STS* (Figure 2D). Ten recombinants were obtained between *M45_STS* and *DGS2*, and six between *M41_STS* and *DGS2* (Table S7 in File S2).

To determine the genomic structure around the *DGS1* and *DGS2* regions, we performed BAC screening for the candidate region of each gene (Figure 2, C and D). For the *DGS1* region, the T65 BAC clones GN0025A01 and GN0028G14 and the *O. nivara* clone IRGC105715_0023B24 were isolated from BAC libraries by using DNA markers *RM16855*, *RM16862*, *RM16867*, and *RM16882*. For the BAC clones around the *DGS2* region, T65 clone GN0031H20 and *O. nivara* clones IRGC105715_0046I04 and IRGC105715_0022L03 were identified by using DNA markers *RM6574*, *M45_STS*, *M41_STS*, and *M23_STS*. Sequencing of one of the *O. nivara* BAC clones at *DGS2*, IRGC105715_0046I04, revealed five tandem copies of a duplicated segment (14,747, 15,902, 14,462, 15,942, and 15,925 bp) in the “finishing-phase” assembly (Figure 2E). Interestingly, these duplicated segments of ∼14–15 kb showed homology to BAC clones covering the regions responsible for *DGS1-T65^+^* as one copy (13,677 bp) and *DGS1-nivara^s^* as one copy (15,968 bp), whereas the duplicated segment was not found (or had been deleted) in BAC clones from the sterile allele *DGS2-T65^s^* (Figure 2E, black arrowhead). We assumed a working hypothesis that the sterility could possibly be explained by the GDRL model at the *DGS1* and *DGS2* loci, which was caused by loss of function of a duplicated gene in the interchromosomal duplicated segment at *DGS1-nivara^s^* and absence of the duplicated gene at *DGS2-T65^s^*. In the segmental duplication on chromosome 4, only one gene, *Os04g0394500*, was predicted in the Rice Annotation Project Database (RAP-DB; http://rapdb.dna.affrc.go.jp). The MSU Rice Genome Annotation Project Database Release 7 (MSU7; http://rice.plantbiology.msu.edu/) also predicted *RPC4* at the same genomic region of *Os04g0394500* as *LOC_Os04g32350*. We conducted 5′- and 3′-RACE reactions to determine the cDNA structure of the *RPC4* homolog of *DGS1-T65^+^* (Figure 2F). The obtained coding sequence of *DGS1-T65^+^* has a different structure from those of *LOC_Os04g32350* and *Os04g0394500* in the second, third, and seventh exons (Figure S3 in File S1). One RNA polymerase III subunit C4 domain (Pfam accession number PF05132) was detected by a Pfam domain search (Finn *et al.* 2014) in the C-terminal region of all three deduced amino acid sequences (Figure 2F and Figure S3 in File S1).

Consistent with the BAC sequencing results, the *DGS1-T65^+^* region contains one copy of the *RPC4* gene, whereas the *DGS2-nivara^+^* region has five tandem copies of *RPC4* (here designated *RPC4a–e*; Figure 2, E and F), whose deduced protein sequences are identical except at the 35th and 225th amino acid residues. At the 35th amino acid residue, the fertile allele *DGS1-T65^+^*, the sterile allele *DGS1-nivara^s^*, and three of the five tandem copies in *DGS2-nivara^+^* possess an isoleucine (I) residue. At the 225th amino acid residue, all of the sequences except one of the five tandem copies in *DGS2-nivara^+^* have a glycine (G) residue. Thus, the observed amino acid substitutions among the deduced proteins were not clearly associated with the allelic functionalities (*i.e.*, fertile or sterile).

To confirm the functionality of the predicted *RPC4* copies, we prepared genomic constructs containing individual *RPC4* genes from the BAC clones of the *DGS1-T65^+^* and *DGS2-nivara^+^* alleles (Figure 2E) and transformed them into *DGS1* semisterile plants (Table 4) and *DGS2* semisterile plants (Table 5). For the transformation of *RPC4* copies from *DGS2-nivara^+^*, we used the *RPC4c*, *RPC4d* (identical to *RPC4b*), and *RPC4e* (identical to *RPC4a*) copies (Figure 2F). Since sterile pollen grains were predicted to carry sterile alleles at both loci, *i.e.*, *DGS1-nivara^s^* and *DGS2-T65^s^*, we hypothesized that introducing a copy of an *RPC4* gene from either of the functional alleles (*DGS1-T65^+^* or *DGS2-nivara^+^*) would rescue the fertility of pollen grains containing it and that *N^s^/N^s^*|*T^s^/T^s^* plants would segregate in the T_1_ generation.

**Table 4:** Segregation of genotypes in T_1_ generation derived by transformation of *RPC4* genes derived from normal alleles into *DGS1* semisterile plants

Transgene (Allele)	Segregation at *RM16862* in T_1_ Generation				*P**^a^*
	*T^+^/T^+^|T^s^/T^s^*	*T^+^/N^s^|T^s^/T^s^*	*N^s^/N^s^|T^s^/T^s^*	Total	
*RPC4* (*DGS1-T65^+^*)	19	39	10	68	8.0 × 10^−4^
*RPC4c* (*DGS2-nivara^+^*)	18	47	15	80	6.7 × 10^−5^
*RPC4d* (*DGS2-nivara^+^*)	22	33	18	73	1.1 × 10^−6^
*RPC4e* (*DGS2-nivara^+^*)	6	16	6	28	5.5 × 10^−4^
Vector control	45	48	1	94	—

a Probabilities were calculated by Fisher’s exact test. We hypothesized that plants carrying the *N^s^/N^s^|T^s^/T^s^* genotype segregate with a probability of 1/94.

**Table 5:** Segregation of genotypes in T_1_ generation derived by transformation of *RPC4* genes derived from normal alleles into *DGS2* semisterile plants

Transgene (Allele)	Segregation at *M41_STS* in T_1_ Generation			
	*N^s^/N^s^*|*T^s^/T^s^*	*N^s^/N^s^*|*N^+^T^s^*	*N^s^/N^s^|N^+^N^+^*	Total
*RPC4* (*DGS1-T65^+^*)	8	21	12	41
*RPC4c* (*DGS2-nivara^+^*)	3	17	6	26
*RPC4d* (*DGS2-nivara^+^*)	6	15	7	28
*RPC4e* (*DGS2-nivara^+^*)	17	37	15	69
Vector control	0	26	22	48

In the T_1_ population derived from transformation of an empty vector into *DGS1* semisterile plants, *T^+^/T^+^*|*T^s^/T^s^*, *T^+^/N^s^*|*T^s^/T^s^*, and *N^s^/N^s^*|*T^s^/T^s^* plants segregated in a 45:48:1 ratio at SSR marker *RM16862*. The single plant with an *N^s^/N^s^*|*T^s^/T^s^* genotype was assumed to be a recombinant between *DGS1* and *RM16862*. *N^s^/N^s^*|*T^s^/T^s^* plants were recovered at significantly higher frequencies in the T_1_ progeny derived from transformation of the *RPC4* genomic segment of *DGS1-T65^+^* into *DGS1* semisterile plants than in the vector control plants (Table 4). To examine the functional redundancy of *RPC4* copies at *DGS1-T65^+^* and *DGS2-nivara^+^*, we also transformed genomic segments containing *RPC4c*, *RPC4d*, and *RPC4e* of the *DGS2-nivara^+^* allele into *DGS1* semisterile plants (Table 4). Similarly, we obtained many *N^s^/N^s^*|*T^s^/T^s^* plants in all T_1_ progeny, at a frequency significantly higher than in the vector control. Likewise, the T_1_ generation of *DGS2* semisterile plants transformed with *RPC4* genes showed recovery of *N^s^/N^s^*|*T^s^/T^s^* plants, whereas the T_1_ generation produced by transformation with the empty vector had no *N^s^/N^s^*|*T^s^/T^s^* plants (Table 5). The observed genotypic frequency in the T_1_ progeny fit the expected ratio of genotypes depending on the numbers of introduced transgenes in the T_0_ generation as determined by quantitative PCR (Table S8 and Table S9 in File S2). These data demonstrate that *DGS1* and *DGS2* are duplicate loci encoding a protein homologous to RPC4.

The sterile allele of *DGS2-T65^s^* was found to be a null allele because *RPC4* was absent (Figure 2E). The other sterile allele, *DGS1-nivara^s^*, is suggested to be a loss-of-function allele caused by a deficiency or absence of expression during male gametogenesis. Within the 3000-bp genomic sequence upstream from the initial codon of the *RPC4* genes, the *RPC4* gene of *DGS1*-*nivara^s^* has 2 nt substitutions—one from G to A at −2373 bp (G−2373A) and one from C to T at −2234 bp from the initial codon (C−2234T)—that distinguish it from all of the fertile alleles (Table S10 in File S2). But we could not conclude that these nucleotide substitutions were essential mutations for the sterility of pollen grains carrying genotype *N^s^*|*T^s^*.

### Duplication of RPC4 homologs in rice

Another putative *RPC4* homolog, *LOC_Os01g66580*, was found on rice chromosome 1 by similarity search using BLAST (Altschul *et al.* 1990). The *LOC_Os01g66580* sequence in the T65 genome was identical to that in the rice reference sequence of Nipponbare. To examine the divergence of RPC4 homologs found in the rice genome, a maximum-likelihood inference tree of the RPC4 domain was constructed using the predicted amino acid sequences of RPC4 homologs from rice and other plants (Figure S4 in File S1). The analysis suggests that the RPC4 homologs of Poales or Poaceae species form two monophyletic clades (designated Poaceae1 and Poaceae2) that separated from those of dicots. The RPC4 homologs encoded by *DGS1-T65^+^* and *DGS2-nivara^+^* were classified into the Poaceae1 clade and the LOC_Os01g66580 protein was classified into the Poaceae2 clade. The RPC4 homologs found in the Zingiberales (banana) and Arecales (African oil palm), which represent other commelinid species, were similar to the ones in the Poaceae1 clade but not to those in the Poaceae2 clade, suggesting that the gene duplication leading to the RPC4 homologs that distinguish the Poaceae1 and Poaceae2 groups occurred in an ancestral population of the Poales or Poaceae, and that RPC4 members in the Poaceae1 clade probably retain the canonical RPC4 function in commelinids. The rice RPC4 members encoded by *DGS1-T65^+^* and *DGS2-nivara^+^* formed one monophyletic group in the Poaceae1 clade. This result suggests that interchromosomal segmental duplication (ISD) between chromosomes 4 and 7 and tandem duplication of *RPC4* members at *DGS2-nivara^+^* occurred after divergence of rice from other Poaceae species.

### Spatial and temporal expression of DGS1 and DGS2

**Figure 5:**
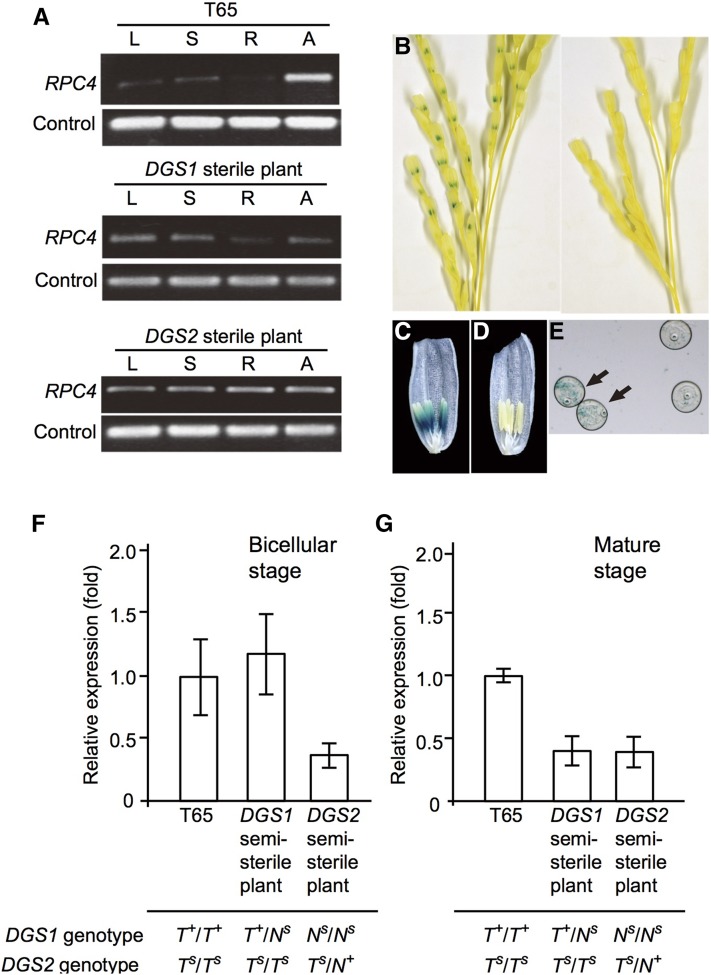
Expression of *DGS1* and *DGS2*. (A) RT-PCR expression analysis of *RPC4* in different plant and tissue types. L, leaf blade; S, leaf sheath; R, roots; A, anther containing pollen at the bicellular stage. Expression was normalized to that of *Ub-CEP52-1* (*Os03g0234200*). (B) Promoter–*GUS* assay of a T_0_ plant transformed with an *RPC4* promoter–*GUS* construct (left) and T65 (right). (C and D) Promoter–*GUS* assay of the floret of a T_0_ plant (C) and T65 (D). (E) Promoter–*GUS* assay of pollen grains from the floret of a T_0_ plant. Arrows indicate blue GUS signal, which segregated in the pollen as expected. (F and G) Relative expression of *RPC4* in anthers of T65, *DGS1* semisterile plants, and *DGS2* semisterile plants, at the (F) bicellular and (G) mature pollen stage. Error bars indicate SEs of three biological replicates (plants). RT-PCR, reverse-transcription polymerase chain reaction.

Expression analysis of *DGS1* and *DGS2* was conducted in the leaf, stem, roots, and anthers of T65 and of *DGS1* and *DGS2* semisterile plants. Because the RNA transcripts derived from different *DGS* genes are almost identical, the expression of those transcripts was analyzed as a whole. Expression of *RPC4* was observed in various tissues, but it was strongest in the anther of T65 (Figure 5A). The expression of *GUS* driven by the *RPC4* promoter derived from the *DGS1-T65^+^* allele was strong in pollen grains, but was not detected in the anther wall or anywhere else (Figure 5, B–E).

By using qRT-PCR, we estimated the *RPC4* transcript levels in anthers of T65, *DGS1* semisterile plants, and *DGS2* semisterile plants (Figure 5, F–G). In the *DGS1* semisterile plants, the *RPC4* transcript level was reduced by half at the mature stage compared with the T65 control (Figure 5G). T65 is homozygous for *DGS1*-*T65^+^*, encoding functional RPC4, and homozygous for *DGS2*-*T65^s^*, from which the *RPC4* gene is completely absent (Figure 2E). T65 produces all normal pollen grains carrying the *T^+^*|*T^s^* genotype, whereas the *DGS1* semisterile plants produce normal pollen grains carrying *T^+^*|*T^s^* and sterile pollen grains carrying *N^s^*|*T^s^*. Therefore, we considered that the reduction in *RPC4* transcript level was probably caused by the 50% reduction in the number of normal pollen grains carrying *T^+^*|*T^s^* in the *DGS1* semisterile plants (*T^+^*/*N^s^*|*T^s^*/*T^s^*) compared with T65 (*T^+^*/*T^+^*|*T^s^*/*T^s^*), and that *DGS1-nivara^s^* was expressed weakly or not at all at the mature stage (Figure 5G). The *DGS2* semisterile plants (*N^s^/N^s^*|*T^s^/N^+^* genotype) also showed half the amount of *RPC4* transcripts compared with T65. These results demonstrate that *RPC4* of *DGS2-nivara^+^* and *RPC4* of *DGS1-T65^+^* express at similar levels because, as previously described, the sterile alleles at each locus appear to contribute little if any RPC4 transcript at the mature stage.

At the bicellular stage, the amount of *RPC4* transcript in the *DGS2* semisterile plants was approximately one-third of that in the T65 control, whereas the amounts of *RPC4* transcripts in the *DGS1* semisterile plants and T65 were comparable (Figure 5F). In *Arabidopsis*, it is suggested that male gametophytes have two sources of PolIII subunits: PolIII machinery synthesized by the diploid meiocyte (sporophyte) and persisting in mature pollen, and machinery synthesized in the developing pollen (gametophyte) (Onodera *et al.* 2008). As one possibility, *RPC4* transcripts synthesized in meiocytes might contribute substantially to the total *RPC4* transcripts in pollen in the *DGS1* semisterile plants at the bicellular stage, since pollen carrying the sterile genotype *N^s^*|*T^s^* did not show any abnormal phenotype. The PolIII machinery synthesized in zygotic meiocytes seems to be enough for pollen maturation and pollen tube germination, because pollen grains carrying the loss-of-function allele for the PolIII subunit complete pollen maturation and pollen tube germination in *Arabidopsis* mutants. However, the mutant *Arabidopsis* pollen did not complete pollen tube elongation because *de novo* synthesis of PolIII machinery is required for late stages of pollen tube elongation (Onodera *et al.* 2008). We speculate that the timing of starvation of PolIII machinery synthesized in the meiocyte is critical for *N^s^*|*T^s^* pollen grains prior to their developmental failure. The sterile pollen grains produced by *DGS1* semisterile plants accomplish starch glycosylation at flowering but fail to germinate, whereas those produced by *DGS2* semisterile plants fail starch glycosylation as early as the preflowering stage (Figure 3, G–L). Although the evidence at this point is indirect, we assume that *DGS1* semisterile plants maintain the same *RPC4* transcript levels as T65 up through the bicellular stage and, thus, achieve further development of pollen grains carrying the sterile genotype *N^s^*|*T^s^* than do *DGS2* semisterile plants. However, all *N^s^*|*T^s^* pollen grains eventually fail in pollen tube germination owing to little or no *de novo RPC4* transcription in pollen and starvation of *RPC4* transcripts synthesized in meiocytes of both *DGS1* and *DGS2* semisterile plants.

## Discussion

### Duplication and loss-of-function of RPC4 caused hybrid pollen sterility

We revealed that a combination of loss-of-function alleles of RPC4 in male gametophytes in hybrids caused hybrid pollen sterility in an interspecific rice hybrid between *O. sativa* ssp. *japonica*-type cultivar T65 and wild *O. nivara*. Pollen sterility was observed in pollen grains carrying two sterile alleles, *DGS1-nivara^s^* and *DGS2-T65^s^* (pollen genotype *N^s^*|*T^s^*). The *DGS2-T65^s^* allele did not possess an *RPC4* copy (Figure 2). For the absence of the *RPC4* copy at *DGS2*-*T65^s^*, two hypotheses were suggested; (1) the *RPC4* copy of the Poaceae1 group was originally located at *DGS1* on chromosome 4 in genus *Oryza* and an ISD did not occur on the ancestral lineage of T65 at *DGS2* on chromosome 7; or (2) the *DGS2*-*T65^s^* allele is the result of an ISD followed by a deletion. It is possible that these hypotheses can be tested by diversity analysis in *O. sativa* and *O. rufipogon* gene pools to investigate the presence or absence of the ISD at *DGS2*. On the other hand, the *RPC4* gene in *DGS1*-*nivara^s^* seemed to contribute little to no *RPC4* transcript in pollen grains carrying the *N^s^*|*T^s^* genotype (Figure 5, F and G). Two nucleotide substitutions, G−2373A and C−2234T, found in a region 3000 bp upstream from the deduced initial codon of the *RPC4* homolog in *DGS1-nivara^s^*, are suggested to be causal variations for nonfunctionalization of the *RPC4* homolog in *DGS1*-*nivara^s^* (Table S10 in File S2). Using genome editing technology, substitution of the “G” nucleotide at position −2373 in functional *RPC4* alleles with the “A” found in *DGS1-nivara^s^* would confirm whether this substitution is required for pollen sterility.

### Male-biased sterility due to lack of an RPC4 homolog in rice

Since RNAPs are vital and fundamental transcriptional machineries in all eukaryotes, a deficit of functional RNAP components would lead to life cycle failure. In this study, gametes carrying the *N^s^*|*T^s^* genotype showed male-biased sterility: male gametophytes carrying both sterile alleles (*N^s^*|*T^s^*) failed in either pollen maturation (*DGS2* semisterile plants) or pollen tube germination (*DGS1* semisterile plants), but there was no obvious abnormality on the female side. Sex-biased sterility in RNAP subunit-deficient mutants has also been observed in *Arabidopsis thaliana*. In mutant lines deficient in the second largest subunits of RNAP I (PolI), II (PolII), and III (PolIII) (designated NRPA2, NRPB2, and NRPC2, respectively), the female gametes carrying defective alleles *nrpa2*, *nrpb2*, or *nrpc2* completely aborted and male gametes with the defective alleles partially retained fertilization ability (Onodera *et al.* 2008). The female-biased sterility in these mutants was suggested to be caused by different characteristics of RNAP synthesis in male and female gametes: the female gamete is autonomous from sporophytic tissues with regard to gene expression and its RNAP is synthesized in the haploid female gametes *de novo*, whereas male gametophytes contain both RNAP machinery that is synthesized in the diploid meiocyte and partitioned into four microspores and machinery newly synthesized in haploid pollen during pollen maturation.

To explain the male-biased sterility observed in this study, we suggest two hypotheses. One is subfunctionalization of DGS1/DGS2 and LOC_Os01g66580 with respect to male *vs.* female gametogenesis. If *de novo* synthesis of RPC4 is indispensable in female gametogenesis in rice, perhaps the RPC4 homolog *LOC_Os01g66580* (chromosome 1) is active in female gametes, thus preserving female fertility. The other hypothesis suggests that the G−2373A nucleotide substitution at *DGS1-nivara^s^* results in loss of transcriptional control of *RPC4* in male gametes only. Analysis of a mutant deficient for *LOC_Os01g66580* would provide better understanding of sex-biased sterility in RPC4 in rice.

### Evolution of transcriptional machinery via gene duplication

Our phylogenetic analysis demonstrated that divergence of the *RPC4* homologs *DGS1*/*DGS2* (Poaceae1) and *LOC_Os01g66580* (Poaceae2) occurred in an ancestral population of the Poales or Poaceae (Figure S4A in File S1), and that the *RPC4* duplication giving rise to *DGS1* and *DGS2* occurred in an ancestral lineage of *O. sativa* and *O. nivara*. Although duplicated genes rapidly lose their function in general, the RPC4 members of the Poaceae2 group have been well conserved in all Poaceae members used in the analysis. RPC4 members in Poaceae2 may have the diverged role in RNA synthesis whereas the Poaceae1 group sequences retain the canonical RPC4 function.

The ancestral lineage of the Poaceae family, such as the BEP clade (rice and purple false brome) and the PACMAD clade (sorghum, maize, foxtail millet, and switchgrass), experienced at least two instances of whole-genome duplication (WGD; paleopolyploidization), called σ and ρ, following the divergence of commelinids (Paterson *et al.* 2004; Wang *et al.* 2005; Tang *et al.* 2010). It has been estimated that the most recent paleopolyploidization, ρ, occurred 70 MYA and that σ paleopolyploidization occurred after the Poaceae–Arecaceae split 120–83 MYA, before divergence of the major Poaceae crop species (Paterson *et al.* 2004, 2009). Arecaceae (African oil palm), Zingiberales (banana), and Asparagales (Crocus) species do not have RPC4 members in Poaceae2 (Figure S4 in File S1). The genus *Oryza* diverged 8–14 MYA (Guo and Ge 2005) and diversification of the AA-genome species occurred over the past 2 MY (Zhu and Ge 2005). Thus, we estimate that the gene duplication leading to *DGS1*/*DGS2* and *LOC_Os01g66580* occurred after the Poaceae–Arecaceae split 120–83 MYA, and that duplication of *RPC4* leading to *DGS1* and *DGS2* occurred > 2–14 MYA. Tang *et al.* (2010) found homeologous chromosomal regions on chromosomes 1 and 4 that originated from the σ paleopolyploidization. However, we do not have sufficient evidence to conclude that the *RPC4* homologs *DGS1*/*DGS2* and *LOC_Os01g66580* were derived from WGD. The other possibility is that the gene duplication leading to *DGS1*/*DGS2* (Poaceae1) and *LOC_Os01g66580* (Poaceae2) resulted from segmental genomic duplication independent from the σ and ρ paleopolyploidization in the ancestral population of the Poaceae.

Segmental duplication and WGD are primary sources of gene redundancy, consequent massive gene losses, and evolutionary novelty (Ohno 1970; Lynch and Conery 2000; Moore and Purugganan 2005). WGD events are found to have independently and sporadically occurred in many angiosperm lineages (James and Lyons 2015). The number of *RPC4* copies observed in this study was fewer than expected based on the number of WGD events experienced in each lineage (Figure S4B in File S1). For example, we found only two *RPC4* copies at present in the banana (Zingiberales) genome, despite the fact that this genome experienced three successive rounds of WGD after divergence from the Poales. *Arabidopsis* underwent three rounds of WGD (α, β, and γ) (Bowers *et al.* 2003), but *A. thaliana* has only two *RPC4* copies, *NPBC14a* and *NPBC14b*, the products of which were proven to have the ability to bind to the PolIII complex in *Arabidopsis* (Ream *et al.* 2015). The phylogenetic analysis showed that the duplication of *NRPC14a* and *NPRC14b* in the Brassicaceae probably originated from a duplication event independent of that related to *RPC4* of the Poaceae1 and Poaceae2 groups. These results suggest that duplicated genes may have been eliminated from gene pools in intermediate evolutionary steps, and positional variations of *RPC4* homologs on chromosomes may have occurred during diploidization after WGDs in several evolutionary lineages, leading to reproductive isolation barriers. In plants, WGD or polyploidization has long been considered a major force of speciation (Stebbins 1950; Soltis *et al.* 2014). Other RNAP components, as well as other duplicated ubiquitous genes indispensable for the life cycle in eukaryotes, may represent potential sources of postzygotic reproductive isolation without any acquisition of functional novelty. In crop species including rice, high-throughput and comprehensive investigation of sequence variants among many varieties and related wild species has actively progressed. This massively accumulated information reveals the numbers, positions, and birth-and-death dating of duplicated genes, and consequently the crop-lineage-specific landscape of genome complexity and plasticity in the history of monocotyledons.

## Supplementary Material

Supplemental material is available online at www.g3journal.org/lookup/suppl/doi:10.1534/g3.117.043943/-/DC1.

## References

[bib1] Altschul S. F. Gish W. Miller W. Myers E. W. Lipman D. J., 1990 Basic local alignment search tool. J. Mol. Biol. 1215: 403–410. 10.1016/S0022-2836(05)80360-2 2231712

[bib2] Bikard D. Patel D. Mette L. C. Giorgi V. Camilleri C., 2009 Divergent evolution of duplicate genes leads to genetic incompatibilities within *A. thaliana*. Science 323: 623–626. 19179528 10.1126/science.1165917

[bib3] Bomblies K. Weigel D., 2007 Hybrid necrosis: autoimmunity as a potential gene-flow barrier in plant species. Nat. Rev. Genet. 8: 382–393. 17404584 10.1038/nrg2082

[bib4] Bowers J. E. Chapman B. A. Rong J. Paterson A. H., 2003 Unravelling angiosperm genome evolution by phylogenetic analysis of chromosomal duplication events. Nature 422: 433–438. 12660784 10.1038/nature01521

[bib5] Brideau N. Flores H. Wang J. Maheshwari S. Wang X., 2006 Two Dobzhansky-Muller genes interact to cause hybrid lethality in *Drosophila*. Science 314: 1292–1295. 17124320 10.1126/science.1133953

[bib6] Chang M. T. Neuffer M. G., 1989 Maize microsporogenesis. Genome 32: 232–244.

[bib7] Chen C. Chen H. Lin Y. S. Shen J. B. Shan J. X., 2014 A two-locus interaction causes interspecific hybrid weakness in rice. Nat. Commun. 5: 3357. 24556665 10.1038/ncomms4357 PMC3948059

[bib8] Coyne J. A. Orr H. A., 2004 *Speciation*. Sinauer Associates, Sunderland, MA.

[bib9] Dellaporta S. Wood J. Hicks J., 1983 A plant DNA minipreparation: version II. Plant Mol. Biol. Report. 1: 19–21.

[bib10] Finn R. D. Bateman A. Clements J. Coggill P. Eberhardt R. Y., 2014 Pfam: the protein families database. Nucleic Acids Res. 42: D222–D230. 24288371 10.1093/nar/gkt1223 PMC3965110

[bib11] Fuse T. Sasaki T. Yano M., 2001 Ti-plasmid vectors useful for functional analysis of rice genes. Plant Biotechnol. 18: 219–222.

[bib12] Guo Y. L. Ge S., 2005 Molecular phylogeny of *Oryzeae* (*Poaceae*) based on DNA sequences from chloroplast, mitochondrial, and nuclear genomes. Am. J. Bot. 92: 1548–1558. 21646172 10.3732/ajb.92.9.1548

[bib13] Haag J. R. Pikaard C. S., 2011 Multisubunit RNA polymerases IV and V: purveyors of non-coding RNA for plant gene silencing. Nat. Rev. Mol. Cell Biol. 12: 483–492. 21779025 10.1038/nrm3152

[bib14] James, S., and E. Lyons, 2015 Plant paleopolyploidy. Figshare. Available at: https://figshare.com/articles/Plant_Paleopolyploidy/1538627.

[bib15] Lander E. S. Green P. Abrahamson J. Barlow A. Daly M. J., 1987 MAPMAKER: an interactive computer package for constructing primary genetic linkage maps of experimental and natural populations. Genomics 1: 174–181. 3692487 10.1016/0888-7543(87)90010-3

[bib16] Long Y. Zhao L. Niu B. Su J. Wu H., 2008 Hybrid male sterility in rice controlled by interaction between divergent alleles of two adjacent genes. Proc. Natl. Acad. Sci. USA 105: 18871–18876. 19033192 10.1073/pnas.0810108105 PMC2596266

[bib17] Lynch M. Conery J. S., 2000 The evolutionary fate and consequences of duplicate genes. Science 290: 1151–1155. 11073452 10.1126/science.290.5494.1151

[bib18] Lynch M. Force A. G., 2000 The origin of interspecific genomic incompatibility via gene duplication. Am. Nat. 156: 590–605. 10.1086/316992 29592543

[bib19] Mann C. Micouin J. Y. Chiannilkulchai N. Treich I. Buhler J. M., 1992 *RPC53* encodes a subunit of *Saccharomyces cerevisiae* RNA polymerase C (III) whose inactivation leads to a predominantly G1 arrest. Mol. Cell. Biol. 12: 4314–4326. 1406624 10.1128/mcb.12.10.4314-4326.1992 PMC360355

[bib20] Mizuta Y. Harushima Y. Kurata N., 2010 Rice pollen hybrid incompatibility caused by reciprocal gene loss of duplicated genes. Proc. Natl. Acad. Sci. USA 107: 20417–20422. 21048083 10.1073/pnas.1003124107 PMC2996679

[bib21] Moore R. C. Purugganan M. D., 2005 The evolutionary dynamics of plant duplicate genes. Curr. Opin. Plant Biol. 8: 122–128. 15752990 10.1016/j.pbi.2004.12.001

[bib22] Nakagawa T. Kurose T. Hino T. Tanaka K. Kawamukai M., 2007 Development of series of gateway binary vectors, pGWBs, for realizing efficient construction of fusion genes for plant transformation. J. Biosci. Bioeng. 104: 34–41. 17697981 10.1263/jbb.104.34

[bib23] Nguyen G. N. Hailstones D. L. Wilkes M. Sutton B. G., 2010 Role of carbohydrate metabolism in drought-induced male sterility in rice anthers. J. Agron. Crop Sci. 196: 346–357.

[bib24] Noor M. A. F. Feder J. L., 2006 Speciation genetics: evolving approaches. Nat. Rev. Genet. 7: 851–861. 17033626 10.1038/nrg1968

[bib25] Ohno S., 1970 *Evolution by Gene Duplication*. Springer, Berlin, Heidelberg, Germany.

[bib26] Onodera Y. Haag J. R. Ream T. Costa Nunes P. Pontes O., 2005 Plant nuclear RNA polymerase IV mediates siRNA and DNA methylation-dependent heterochromatin formation. Cell 120: 613–622. 15766525 10.1016/j.cell.2005.02.007

[bib27] Onodera Y. Nakagawa K. Haag J. R. Pikaard D. Mikami T., 2008 Sex-biased lethality or transmission of defective transcription machinery in *Arabidopsis*. Genetics 180: 207–218. 18723889 10.1534/genetics.108.090621 PMC2535675

[bib28] Paterson A. H. Bowers J. E. Chapman B. A., 2004 Ancient polyploidization predating divergence of the cereals, and its consequences for comparative genomics. Proc. Natl. Acad. Sci. USA 101: 9903–9908. 15161969 10.1073/pnas.0307901101 PMC470771

[bib29] Paterson A. H. Bowers J. E. Bruggmann R. Dubchak I. Grimwood J., 2009 The *Sorghum bicolor* genome and the diversification of grasses. Nature 457: 551–556. 19189423 10.1038/nature07723

[bib30] Ream T. S. Haag J. R. Wierzbicki A. T. Nicora C. D. Norbeck A., 2009 Subunit compositions of the RNA-silencing enzymes pol IV and pol V reveal their origins as specialized forms of RNA polymerase II. Mol. Cell 33: 192–203. 19110459 10.1016/j.molcel.2008.12.015 PMC2946823

[bib31] Ream T. S. Haag J. R. Pontvianne F. Nicora C. D. Norbeck A. D., 2015 Subunit compositions of Arabidopsis RNA polymerases I and III reveal Pol I- and Pol III-specific forms of the AC40 subunit and alternative forms of the C53 subunit. Nucleic Acids Res. 43: 4163–4178. 25813043 10.1093/nar/gkv247 PMC4417161

[bib32] Rieseberg L. H. Blackman B. K., 2010 Speciation genes in plants. Ann. Bot. 6: 439–455. 10.1093/aob/mcq126 PMC2924826 20576737

[bib33] Soltis D. E. Visger C. J. Soltis P. S., 2014 The polyploidy revolution then…and now: Stebbins revisited. Am. J. Bot. 101: 1057–1078. 25049267 10.3732/ajb.1400178

[bib34] Stebbins G. L., 1950 *Variation and Evolution in Plants*. Oxford University Press, London, UK.

[bib35] Tang H. J. E. Bowers X. Wang A. H. Paterson A. H., 2010 Angiosperm genome comparisons reveal early polyploidy in the monocot lineage. Proc. Natl. Acad. Sci. USA 107: 472–477. 19966307 10.1073/pnas.0908007107 PMC2806719

[bib36] Taylor J. S. Van de Peer Y. Meyer A., 2001 Genome duplication, divergent resolution and speciation. Trends Genet. 17: 299–301. 11377777 10.1016/s0168-9525(01)02318-6

[bib37] Temnykh S. DeClerck G. Lukashova A., 2001 Computational and experimental analysis of microsatellites in rice (*Oryza sativa* L.): frequency, length variation, transposon associations, and genetic marker potential. Genome Res. 11: 1441–1452. 11483586 10.1101/gr.184001 PMC311097

[bib38] Vannini A. Cramer P., 2012 Conservation between the RNA polymerase I, II, and III transcription initiation machineries. Mol. Cell 45: 439–446. 22365827 10.1016/j.molcel.2012.01.023

[bib39] Wang X. Shi X. Hao B. Ge S. Luo J., 2005 Duplication and DNA segmental loss in the rice genome: implications for diploidization. New Phytol. 165: 937–946. 15720704 10.1111/j.1469-8137.2004.01293.x

[bib40] Wang Y. Ma H., 2015 Step-wise and lineage-specific diversification of plant RNA polymerase genes and origin of the largest plant-specific subunits. New Phytol. 207: 1198–1212. 25921392 10.1111/nph.13432

[bib41] Win K. T. Yamagata Y. Miyazaki Y. Doi K. Yasui H., 2011 Independent evolution of a new allele of F_1_ pollen sterility gene *S27* encoding mitochondrial ribosomal protein L27 in *Oryza nivara*. Theor. Appl. Genet. 122: 385–394. 20878142 10.1007/s00122-010-1454-y

[bib42] Yamagata Y. Yamamoto E. Aya K. Win K. T. Doi K., 2010 Mitochondrial gene in the nuclear genome induces reproductive barrier in rice. Proc. Natl. Acad. Sci. USA 107: 1494–1499. 20080642 10.1073/pnas.0908283107 PMC2824375

[bib43] Yamamoto E. Takashi T. Morinaka Y. Lin S. Wu J., 2010 Gain of deleterious function causes an autoimmune response and Bateson–Dobzhansky–Muller incompatibility in rice. Mol. Genet. Genomics 283: 305–315. 20140455 10.1007/s00438-010-0514-y

[bib44] Yang J. Zhao X. Cheng K. Du H. Ouyang Y., 2012 A killer-protector system regulates both hybrid sterility and segregation distortion in rice. Science 337: 1336–1340. 22984070 10.1126/science.1223702

[bib45] Zhu Q. Ge S., 2005 Phylogenetic relationships among A-genome species of the genus *Oryza* revealed by intron sequences of four nuclear genes. New Phytol. 167: 249–265. 15948847 10.1111/j.1469-8137.2005.01406.x

