# Supplementary material for: Duplication and Loss of Function of Genes Encoding RNA Polymerase III Subunit C4 Causes Hybrid Incompatibility in Rice
Source: G3 (Bethesda). 2017 Jun 7;7(8):2565–75. doi: 10.1534/g3.117.043943 (PMC5555463; doi:10.1534/g3.117.043943)
Supplement: Supplementary file 1 [file 2565FileS1.docx]

**Duplication and Loss of Function of Genes Encoding RNA Polymerase III Subunit C4 Causes Hybrid Incompatibility in Rice**

Giao Ngoc Nguyen^*,1^, Yoshiyuki Yamagata^*,1^, Yuko Shigematsu^*^, Miyako Watanabe^*^, Yuta Miyazaki^*^, Kazuyuki Doi^*,2^, Kosuke Tashiro^†^, Satoru Kuhara^†^, Hiroyuki Kanamori^‡^, Jianzhong Wu,^‡^ Takashi Matsumoto,^‡^ Hideshi Yasui,* Atsushi Yoshimura^*,3^

Author affiliations:

^*^Plant Breeding Laboratory, Faculty of Agriculture, Kyushu University, Fukuoka, Fukuoka 812-8581, Japan

^†^Molecular Gene Technics Laboratory, Faculty of Agriculture, Kyushu University, Fukuoka, Fukuoka 812-8581, Japan

^‡^Agrogenomics Research Center, National Institute of Agrobiological Sciences, 1-2 Ohwashi, Tsukuba, Ibaraki 305-8634 Japan

^1^These authors contributed equally to this work.

^2^Present address: Graduate School of Bioagricultural Sciences, Nagoya University, Chikusa, Nagoya 464-8601, Japan

^3^Corresponding author: Plant Breeding Laboratory, Faculty of Agriculture, Kyushu University, Japan.

**Supplemental Methods**

**Estimated frequency of genotypes in complementation test**

Here we assume one transgenic T_0_ plant carrying *k* transgene insertions that are transmitted to the progeny and sort independently, produced by *Agrobacterium* transformation of a recipient plant heterozygous at *DGS1* and homozygous for the *DGS2-T65^s^* allele (*T^+^/N^s^*|*T^s^*/*T^s^*). In gametes of the T_0_ plants, *k* transgene insertions are expected to segregate into 2*^k^* different genotypes.

Let *f*_+_ and *f_−_* represent the estimated segregation frequency of gametes with at least one transgene (*+*) or without a transgene (−), respectively.

Where *k* is an integer >0,

*f_+_* = 1 − (1/2)*^k^* . (1.1)

And where *k* is an integer >0,

*f_−_* = (1/2)*^k^* . (1.2)

Using this model, we can estimate the frequencies of segregating gametes carrying the genotype *T^+^*|*T^s^* with at least one transgene (*T^+^*|*T^s^*|*+*), gametes carrying the genotype *T^+^*|*T^s^* without any transgene (*T^+^*|*T^s^*|*−*), gametes carrying the genotype *N^s^*|*T^s^* with at least one transgene (*N^s^*|*T^s^*|*+*), and gametes carrying genotype *N^s^*|*T^s^* without any transgene (*N^s^*|*T^s^*|*−*) as 1/2*f_+_*, 1/2*f_−_*, 1/2*f_+_*, and 1/2*f_−_*, respectively. The estimated segregation frequencies in progeny of self-pollinated T_0_ plants are represented as *a_i_* (*i* = 1, 2 , ..., 9) in the Punnett square in Table 1. Note that male gametes with the *N^s^*|*T^s^*|*−* genotype are sterile.

Then, we can estimate the frequencies of homozygotes for the *DGS1-T65^+^* allele (*f_T/T, DGS1_*), heterozygotes (*f_T/N, DGS1_*), and homozygotes for the *DGS1-nivara^s^* allele (*f_N/N, DGS1_*) as follows:

*f_T/T, DGS1_* = *a_1_* + *a_2_* + *a_5_* + *a_6_*

= (1/2*f_+_*)^2^ + (1/2*f_+_*)(1/2*f_−_*) + (1/2*f_+_*)(1/2*f_−_*) + (1/2*f_−_*)^2^

= 1/4 (2.1)

*f_T/N, DGS1_* = *a_3_* + *a_4_* + *a_7_* + *a_8_* + *a_9_* + *a_10_*

= (1/2*f_+_*)^2^ + (1/2*f_+_*)(1/2*f_−_*) + (1/2*f_+_*)(1/2*f_−_*) + (1/2*f_+_*)^2^ + (1/2*f_+_*)(1/2*f_−_*)

= 1/2 − (1/2)*^k+2^* (2.2)

*f_N/N, DGS1_* = *a_11_* + *a_12_*

= (1/2*f_+_*)(1/2*f_−_*) + (1/2*f_−_*)^2^

= 1/4 − (1/2)*^k+2^*. (2.3)

Therefore, when *k* = 1, the estimated ratio of *T^+^*/*T^+^* : *T^+^*/*N^s^* : *N^s^*/*N^s^* at *DGS1* is 2:3:1, and when *k* = 5, it is 32:63:31.

Similarly, when the recipient plant is heterozygous at *DGS2* and homozygous for the *DGS1-nivara^s^* allele, the frequencies of homozygotes for the *DGS2-T65^s^* allele (*f_T/T, DGS2_*), heterozygotes (*f_T/N, DGS2_*), and homozygotes for the *DGS2-nivara^+^* allele (*f_N/N, DGS2_*) at *DGS2* are estimated as

*f_T/T, DGS2_* = 1/4 − (1/2)*^k^*^+2^, (3.1)

*f_T/N, DGS2_* = 1/2 − (1/2)*^k^*^+2^, (3.2)

and *f_N/N, DGS2_* = 1/4 . (3.3)

Thus, when *k* = 1, the estimated ratio of *T^s^*/*T^s^* : *T^s^*/*N^+^* : *N^+^*/*N^+^* at *DGS2* = 1:3:2, and when *k* = 4, it is 15:31:16.

**Construction of phylogenetic tree**

The DGS1-T65 sequence was used in a BLASTP search of proteome databases in the plant comparative genomics database Phytozome (http://phytozome.jgi.doe.gov/pz/portal.html) with a cutoff E-value of 1, and all homologous protein entries were downloaded from the site. RNA polymerase III subunit C4 domains (RNA_pol_Rpc4, Pfam accession number PF05132, Pfam http://pfam.xfam.org/) in the downloaded proteins were detected by hidden Markov model searches using *hmmsearch* software (http://hmmer.org/) with a cutoff score of 1e−8. After the amino acid sequences of the RPC4 domains were aligned in MUSCLE software with default parameters (Edgar 2004), a phylogenetic tree based on maximum-likelihood inference was constructed in RAxML v. 8.2.8 software (Stamatakis 2014) and drawn in FigTree v. 1.4.2 software (http://tree.bio.ed.ac.uk/software/figtree/).

**References**

Edgar, R. C., 2004 MUSCLE: a multiple sequence alignment method with reduced time and space complexity. BMC Bioinformatics 5: 113.

Stamatakis, A., 2014 RAxML version 8: A tool for phylogenetic analysis and post-analysis of large phylogenies. Bioinformatics 30: 1312–1313.

**Figure S1** Plant materials in this study. (A) Breeding scheme of the plant materials used in this study. The rice SSR markers shown in (B) were used for marker-assisted selection (MAS) at *DGS1* and *DGS2*. (B) Graphical genotype of the BC_4_F_2_ 42 population. White, T65 homozygous region; grey, heterozygous region for T65 (*O. sativa*) and O. nivara (IRGC105715). Horizontal bars show map locations of SSR markers used for genome-wide genotyping. Physical marker positions on the rice reference sequence of Nipponbare (Os-Nipponbare-Reference-IRGSP-1.0 pseudomolecules) are shown in Table S1.

**Figure S2** Genetic model of the gametophytic type of Bateson–Dobzhansky–Muller (BDM) incompatibility. (A) Phenotypes of pollen grains determined by pollen genotype. Pollen grains carrying only sterile alleles (*DGS1-nivara^s^* and *DGS2-T65^s^*) are sterile, whereas pollen grains carrying at least one fertile allele (*DGS1-T65^+^* or *DGS2-nivara^+^*) are fertile. (B) Punnett square for progeny of self-pollinated plants heterozygous at both *DGS1* and *DGS2*. Since pollen grains carrying genotype *DGS1-IRGC105715^s^* | *DGS2-T65^s^* (*N^s^*|*T^s^*) are not fertile, the nine possible genotypes (*T^+^/T^+^*|*T^s^/T^s^* : *T^+^/T^+^*|*T^s^/N^+^* : *T^+^/T^+^*|*N^+^/N^+^* : *T^+^/N^s^*|*T^s^/T^s^* : *T^+^/N^s^*|*T^s^/N^+^* : *T^+^/N^s^*|*N^+^/N^+^* : *N^s^/N^s^*|*T^s^/T^s^* : *N^s^/N^s^*|*T^s^/N^+^* : *N^s^/ N^s^*|*N^+^/N^+^*) are predicted to segregate in a 1:2:1:1:3:2:0:1:1 ratio. (C) Three phenotypic classes are expected to segregate in progeny of self-pollinated plants heterozygous at both *DGS1* and *DGS2*. Plants showing 100%, 75%, and 50% pollen fertility are expected to segregate in a 7:3:2 ratio.

**Figure S3** Comparison of gene structure of *DGS1* between gene models reported in publicly available rice annotation databases and the *DGS1-T65* gene structure examined by 5′ and 3′ rapid amplification of cDNA ends (RACE). The *DGS1-T65* cDNA sequence was examined by 5′ and 3′ RACE reactions (GenBank accession no. AB758279). The *LOC_Os04g32350* gene structure information was obtained from MSU Rice Genome Annotation Project Release 7 (MSU7, http://rice.plantbiology.msu.edu/). Th e *Os04g0394500* gene structure information was obtained from the Rice Annotation Project Database (RAP-DB) of the International Rice Genome Sequencing Project (http://rapdb.dna.affrc.go.jp/). White boxes indicate untranslated regions; gray boxes indicate coding sequence (CDS) regions; yellow boxes indicate exons with a structure different from the cDNA sequence determined by RACE. The estimated CDS of *LOC_Os04g32350* does not have the exon corresponding to the 3rd exon of the *DGS1-T65* cDNA sequence determined by RACE, and the deduced position of the initial codon of the 2nd exon of *LOC_Os04g32350* was located 34 bp downstream relative to the *DGS1-T65* cDNA sequence. The estimated CDS In *Os04g0394500* does not have the initial codon (truncated) and has a shortened exon corresponding to the 7th exon of the *DGS1-T65* cDNA sequence. In each of the three gene models, the sequence predicted to encode the RNA polymerase III subunit C4 domain was detected by a Pfam domain search (red underlines).

**Figure S4** Phylogenetic analysis of RPC4 domains in plants.

(A) Phylogenetic tree constructed using predicted amino acid sequences of the RNA polymerase III subunit C4 (RPC4) domain region (see Supplemental Methods). Among the RPC4 homologs of the eudicot species, fabids and malvids are indicated in purple and blue, respectively. Brassicaceae homologs are indicated in green. RPC4 homologs of rice and other monocots are indicated in red and orange, respectively. Numbers at nodes are bootstrap values of >750 from 1000 replicates.

(B) Cladogram of species used in the phylogenetic analysis. Taxonomic information of the species was obtained from the National Center for Biotechnology Information (NCBI) Taxonomy browser (https://www.ncbi.nlm.nih.gov/Taxonomy/taxonomyhome.html/index.cgi). Red triangles indicate times when whole-genome duplication (WGD) events are reported to have occurred. Poaceae WGD events rho and sigma (Paterson *et al.* 2004; Wang *et al.* 2005; Tang *et al.* 2010) are indicated by ‘ρ’ and ‘σ’, respectively. *Arabidopsis* WGD events alpha, beta, and gamma (Bowers *et al.* 2003) are indicated by ‘α’, ‘β’, and ‘γ’, respectively.
